# Clinical and Angioarchitectural Risk Factors Associated with Intracranial Hemorrhage in Dural Arteriovenous Fistulas: A Single-Center Retrospective Study

**DOI:** 10.1371/journal.pone.0131235

**Published:** 2015-06-29

**Authors:** Chuanhui Li, Yang Wang, Youxiang Li, Chuhan Jiang, Xinjian Yang, Zhongxue Wu

**Affiliations:** Department of Interventional Neuroradiology, Beijing Neurosurgical Institute and Beijing Tiantan Hospital, Capital Medical University, Beijing, China; Charité Universitätsmedizin Berlin, NeuroCure Clinical Research Center, GERMANY

## Abstract

**Purpose:**

To investigate which clinical and angioarchitectural features were associated with the occurrence of intracranial hemorrhage in patients with intracranial dural arteriovenous fistulas (DAVFs).

**Materials and Methods:**

We retrospectively reviewed the clinical and angioarchitectural features of 236 consecutive patients diagnosed with DAVF in our department from April 2009 to November 2013. Two groups of patients, with or without intracranial hemorrhage as clinical presentation at the initial diagnosis, were analysed to identify the differences in clinical and angioarchitectural features in univariate analysis. A multivariate logistic regression model was also developed to assess the independent contribution of the potential risk factors. Associations were considered significant for p<0.05.

**Results:**

Fifty-six patients (23.7%) presented with intracranial hemorrhage at the initial diagnosis of DAVF. In univariate analysis, male patients (p = 0.002), patients with medical history of smoking (p<0.001) or alcohol consumption (p = 0.022), and DAVFs located at the tentorium (p = 0.010), frontalbasal (p = 0.007), foramen magnum (p = 0.043) or cerebral convexity (p<0.001) were associated with an increased risk of intracranial hemorrhage. A higher risk of hemorrhagic occurrence was also observed in DAVFs with superficial cortical venous drainage (p<0.001), deep venous drainage (p = 0.003), occluded venous sinus (p<0.032), or higher Borden type (p<0.001). A multivariate logistic regression model showed that intracranial hemorrhage in patients with DAVFs was correlated with higher Borden classification (OR 5.880; 95% CI, 3.370–10.257; p<0.001).

**Conclusion:**

Venous drainage pattern was the only independent risk factor of intracranial hemorrhage in our patients with intracranial DAVF. The other potential risk factors may be confounding factors in predicting intracranial hemorrhage.

## Introduction

Intracranial dural arteriovenous fistulas (DAVFs) are special arteriovenous malformations (AVMs) inside the dura that manifest with a variety of clinical presentations, including pulsatile tinnitus, headache, exophthalmos, and sometimes nonhemorrhagic neurologic deficits or intracranial hemorrhage [1, 2]. Intracranial hemorrhage represents one of the most serious clinical manifestations of DAVF, with reported frequency ranging from 12% to 22% [2–5] and poor long-term prognosis resulting from debilitating neurological deficits and rebleeding [3, 6, 7].

Several previous retrospective studies have analyzed the risk factors of intracranial DAVF associated with hemorrhage [2, 4, 5, 7–12]. However, most of these studies focused only on angioarchitectural characteristics [2, 5, 7–12] and some of these studies included only < 100 patients [7–9, 11]. Moreover, some results of these studies are contradictory and controversial. Currently, except for cortical venous drainage, no other factor has been widely recognized as an independent risk factors for hemorrhagic presentation in patients with DAVF. Based on a large series of patients with DAVF, the purpose of this study was to identify the clinical and angioarchitectural features that were associated with the occurrence of intracranial hemorrhage.

## Materials and Methods

Patients or their family members gave written informed consent to participate in this study and to publish related materials. Because of the retrospective nature of the study, informed consent is unavailable from some patients; and family member of the patients consented on their behalf under this circumstance. This retrospective study, including the consent procedure, was approved by Beijing Tiantan Hospital’s ethics committee.

### Patients and data collection

We reviewed our patient database from April 2009 to November 2013 and identified all patients with DAVF meeting the following inclusion and exclusion criteria. The inclusion criteria included: (1) Diagnosis of DAVF was confirmed by conventional cerebral angiograms. (2) The clinical and radiologic data is complete, including computed tomography/magnetic resonance (CT/MR) imaging and preoperative cerebral angiograms. (3) Written informed consent was obtained. Exclusion criteria included: (1) definitively traumatic or iatrogenic DAVFs (because these lesions were thought to be another pathologic process and the natural history may be different from spontaneous DAVFs). (2) The patients harboring coexistence of DAVF and other neurovascular lesions, such as cerebral arteriovenous malformations, intracranial aneurysms, direct carotid cavernous fistula or Moyamoya disease. (3) Patients with multiple DAVFs (4) The clinical or radiologic data is incomplete. (5)The informed consent was unavailable.

Demographic and clinical characteristics were collected by retrospectively reviewing the clinical records, and were further confirmed by phone contact with the patients or their close relatives. We collected data on the following variables: patient age, sex, medical history including hypertension, diabetes mellitus, hyperlipidemia, smoking history and alcohol consumption status, type of nonhemorrhagic clinical presentation (benign or aggressive). Patient age was recorded as the age at diagnosis of DAVF. Hypertension was defined as taking antihypertensive agents, a systolic blood pressure ≥ 140 mm Hg, or a diastolic blood pressure ≥ 90 mm Hg before the onset of intracranial hemorrhage. Diabetes mellitus was defined as taking antidiabetic agents, treatment with insulin injection, a fasting plasma glucose level ≥ 126 mg/dl, a random plasma glucose level > 200 mg/dl, or a hemoglobin A1C (GHbA1C) level ≥ 6.5%. Hyperlipidemia was defined as taking antihyperlipidemic agents, having a total cholesterol (TC) level ≥ 220 mg/dl, a triglyceride (TG) level ≥ 200 mg/dl, or a high density lipoprotein cholesterol (HDL-C) level < 40mg/dl. Patients were coded as positive for smoking if they were current or past tobacco users, for alcohol consumption if they consumed alcohol more than 5 times a week. The nonhemorrhagic clinical presentations were separated into 2 groups: benign or aggressive. The benign group included an incidental finding, nonspecific headaches, chemosis/proptosis, blurred vision, bruit or pulsatile tinnitus and cranial nerve deficits. The aggressive group consisted of seizures, motor or sensory neurologic deficits, cerebellar symptoms, visual field defects or blindness, aphasia, parkinsonism, dementia, and other nonhemorrhagic neurologic deficits.

Angioarchitectural characteristics recorded for this study included location of DAVFs, the number of fistulas (simple fistula or multiple fistulas), fistula flow velocity (high-velocity or low-velocity), origin of the arterial feeders, presence or absence of superficial cortical venous drainage and deep venous drainage, venous drainage pattern (Borden type I, II, or III, Table 1), presence or absence of occluded venous sinus. The angioarchitectural characteristics mentioned above were independently reviewed and evaluated by at least two experienced interventional neuroradiologist, who were blinded to clinical data. Location of DAVFs was classified as: cavernous sinus, transverse & sigmoid sinus, superior sagittal sinus, tentorium, frontalbasal, middle fossa, foramen magnum, cerebral convexity and others. In accordance with the classification system of Daniels et al [1], fistulas were localized to the tentorium if the vein draining the fistulas emerged from the superior or inferior, the area of the tentorial incisura, including the galenic system and the tentorial attachment. As proposed by Borden et al [13], a simple fistula is a direct connection between a single meningeal artery and a draining vein or sinus, while lesions with a more complex structure which are fed by multiple arteries were defined as multiple fistulas. The fistula flow velocity was grouped into two types, high-velocity or low-velocity, according to comparison of the contrast agent filling time of the fistula/venous drainage and that of filling of the distal territory of major feeding arteries. A high-velocity fistula flow rate was defined as the appearance of the DAVF venous phase prior to filling of the distal territory of major feeding arteries. In contrast, low fistula flow rate was defined as an initial contrast agent filling time of the fistula/venous drainage later than filling of the distal territory of major feeding arteries. When the fistula was supplied simultaneously by arterial feeders from 2 or 3 of the internal carotid, external carotid and vertebrobasilar systems, the fistula was defined as type of arterial feeder from multiple systems.

**Table 1:** Borden classification of DAVFs (n = 236).

Borden classification	Definition	No. of patients (%)
type I	drains directly into major venous sinus or meningeal vein	91 (38.6%)
type II	drains into venous sinus but high pressure w/in sinus results also in retrograde drainage via subarachnoid veins	54 (22.9%)
type III	drains directly into subarachnoid veins	91 (38.6%)

In this study, patients were divided into hemorrhagic group and nonhemorrhagic group according to whether the patients experienced intracranial hemorrhage or not at the diagnosis of DAVF. Evidence of intracranial hemorrhage was confirmed with signs of intracranial blood on CT scan or MR imaging, or sometimes in the cerebrospinal fluid by lumbar puncture. For all patients of the hemorrhagic group in this series, the source of intracranial hemorrhage was attributed to the DAVF because there was no other explanation.

### Statistical analysis

All statistical analyses were performed using the software package SPSS 17.0 (SPSS, Chicago, IL, USA). Except for patient age at diagnosis, all the variables were categorical and reported as count and percentage. In univariate analysis, normally distributed quantitative variables were compared using the Student t-test between patients with and without intracranial hemorrhage; and non–normally distributed categorical variables were compared using the Chisquare test. A logistic regression model was used for multivariate analysis to determine if the potential risk factors in univariate analysis remained independently associated with hemorrhagic presentation. The statistical level of significance was p = 0.05.

## Results

### Patient demographics and baseline characteristics

We enrolled 236 patients with DAVF, 153 male (64.8%) and 83 female (35.2%). The age at the time of diagnosis ranged from 10 years to 76 years (mean 48 ± 13.0 years) (Table 2). Clinical manifestations at diagnosis of DAVFs were bruit/pulsatile tinnitus in 130 patients (55.1%), headache in 100 (42.4%), chemosis/exophthalmos in 84 (35.6%), visual deterioration/blindness in 21 (8.9%), seizure in 15 (6.4%), mental deterioration/cognitive decline in 9 (3.8%). Fifty-six patients (23.7%) presented with intracranial hemorrhage, while in 37 patients (15.7%) there were asymptomatic at the time of diagnosis. Location was cavernous sinus in 65 patients (27.5%), transverse & sigmoid sinus in 61 (25.8%), superior sagittal sinus in 15 (6.4%), tentorium in 51 (21.6%), frontalbasal in 21 (8.9%), middle fossa in 4 (1.7%), foramen magnum in 4 (1.7%) and convexity in 13 (5.5%). Demographic, clinical and angioarchitectural characteristics of the patient population are shown in Tables 1 and 2.

**Table 2:** Demographic and clinical features of patient population.

Variables	N (%)
Number of patients	236 (100%)
Sex	
male	153 (64.8%)
female	83 (35.2%)
Age at diagnosis (yrs)	48 ± 13.0
Clinical manifestation at diagnosis	
intracranial hemorrhage	56 (23.7%)
tinnitus/bruit	130 (55.1%)
headache	100 (42.4%)
chemosis/exophthalmos	84 (35.6%)
visual deterioration/blindness	21 (8.9%)
seizure	15 (6.4%)
mental deterioration/cognitive decline	9 (3.8%)
other neurological deficit	10 (4.2%)
asymptomatic	37 (15.7%)
Location of DAVF	
cavernous sinus	65 (27.5%)
transverse & sigmoid sinus	61 (25.8%)
superior sagittal sinus	15 (6.4%)
tentorium	51 (21.6%)
frontalbasal	21 (8.9%)
middle fossa	4 (1.7%)
foramen magnum	4 (1.7%)
convexity	13 (5.5%)
others	2 (0.8%)

### Characteristics of the patients in hemorrhagic group

Fifty-six patients (23.7%) presented with intracranial hemorrhage at diagnosis of DAVF. Forty-six patients (82.1%) were male and 10 (17.9%) female. Mean age at the time of diagnosis was 48 ± 12.0 years (Table 3). Tentorial fistulas (n = 19, 33.9%) were the most common location of DAVF followed by the anterior cranial fossa (n = 10, 17.9%) and transverse & sigmoid sinus (n = 8, 14.3%). Concerning the type of intracranial hemorrhage extensions, 34 patients (60.7%) had intraparenchymal hemorrhage, 13 (23.2%) subarachnoid, 9 (16.1%) intraparenchymal & intraventricular, 2 (3.6%) subdural and 1 (1.8%) intraventricular. As for medical history of the patients in this group, hypertension was present in 16 patients (28.6%), diabetes mellitus in 3 (5.4%), hyperlipidemia in 15 (26.8%), smoking in 30 (53.6%), and alcohol consumption in 24 (42.9%).

**Table 3:** Demographic, clinical and angiographic characteristics of patients with dural arteriovenous fistula in hemorrhagic and nonhemorrhagic groups.

Variables		Hemorrhagic group (n = 56)	Nonhemorrhagic group (n = 180)	P value	P, Regression(OR, 95% CI)
Age (yrs), Mean ± SD		48 ± 12.0	48 ± 13.4	0.994	
Age > 50 yrs		24 (42.9%)	79 (43.9%)	0.892	
Male		46 (82.1%)	107 (59.4%)	**0.002**	0.838 (1.115, 0.392–3.175)
Aggressive nonhemorrhagic clinical presentation		8 (14.3%)	32 (17.8%)	0.543	
Medical history					
	hypertension	16 (28.6%)	51 (28.3%)	0.972	
	diabetes mellitus	3 (5.4%)	24 (13.3%)	0.101	
	hyperlipidemia	15 (26.8%)	31 (17.2%)	0.115	
	smoking	30 (53.6%)	41 (22.8%)	**<0.001**	0.096 (1.868, 0.896–3.896)
	alcohol consumption	24 (42.9%)	48 (26.7%)	**0.022**	0.326 (1.538, 0.651–3.631)
Location of DAVF					
	cavernous sinus	1 (1.8%)	64 (35.6%)	**<0.001**	0.522 (0.473, 0.048–4.679)
	transverse & sigmoid sinus	8 (14.3%)	53 (29.4%)	**0.024**	0.623 (0.718, 0.191–2.695)
	superior sagittal sinus	4 (7.1%)	11 (6.1%)	1.000	
	tentorium	19 (33.9%)	32 (17.8%)	**0.010**	0.719 (0.774, 0.193–3.111)
	frontalbasal	10 (17.9%)	11 (6.1%)	**0.007**	0.280 (1.886, 0.596–5.962)
	middle fossa	1 (1.8%)	3 (1.7%)	1.000	
	foramen magnum	3 (5.4%)	1 (0.6%)	**0.043**	0.101 (12.703, 0.607–265.645)
	convexity	9 (16.1%)	4 (2.2%)	**<0.001**	0.121 (2.751, 0.766–9.878)
	others	1 (1.8%)	1 (0.6%)	0.419	
Multiple Fistulas		45 (80.4%)	158 (87.8%)	0.162	
High fistula flow velocity		47 (83.9%)	163 (90.6%)	0.167	
Bilateral arterial feeders		23 (41.1%)	86 (47.8%)	0.379	
Arterial feeder from multiple systems		38 (67.9%)	149 (82.8%)	**0.016**	0.869 (1.080, 0.431–2.707)
Superficial cortical venousdrainage		44 (78.6%)	73 (40.6%)	**<0.001**	0.289 (1.583, 0.678–3.698)
Deep venous drainage		18 (32.1%)	26 (14.4%)	**0.003**	0.084 (2.271, 0.895–5.760)
Occluded venous sinus		13 (23.2%)	21 (11.7%)	**0.032**	0.058 (2.416, 0.970–6.018)
Venous drainage pattern				**<0.001**	**<0.001 (5.880, 3.370–10.257)**
	Borden type I	2 (3.6%)	89 (49.4%)		
	Borden type II	9 (16.1%)	45 (25.0%)		
	Borden type III	45 (80.4%)	46 (25.6%)		

The DAVFs in 38 patients (67.9%) had arterial feeders from multiple systems, and 23 of them were fed by bilateral arterial feeders. The fistula flow velocity was high in 47 patients (83.9%). The DAVFs was single type in 11 patients (19.6%), while multiple fistulas were observed in 45 (80.4%). Concerning the venous drainage pattern, the DAVFs in 2 patients (3.6%) were Borden type I, 9 (16.1%) Borden type II, 45 (80.4%) Borden type III. Superficial cortical venous drainage was present in 44 patients (78.6%), while deep venous drainage in 18 (32.1%) patients. Occluded venous sinus was observed in 13 (23.2%) patients.

### Statistical analysis

Results of statistical analysis were summarized in Table 3.

For demographic and clinical characteristics, univariate statistical analysis showed no difference regarding the patient age (whether the original age or patient age dichotomized by the point of 50 years, p = 0.994 and p = 0.892 respectively) between the hemorrhagic and nonhemorrhagic group. On the contrary, we found a significant difference concerning sex. Male patients were associated with increased risk of intracranial hemorrhage (82.1% vs 59.4%, p = 0.002). Among the variables concerning medical history, history of smoking and alcohol consumption were both significantly associated with higher risk of intracranial hemorrhage (53.6% vs 22.8%, p<0.001; 42.9% vs 26.7%, p = 0.022, respectively), while no significant relationship was observed between the other 3 variables and bleeding occurrence. Moreover, there was no significant difference for the type of nonhemorrhagic clinical presentation between the 2 groups (p = 0.543).

DAVFs located at the cavernous sinus and transverse & sigmoid sinus were associated with a significantly lower risk of intracranial hemorrhage when compared with the lesions located at other regions (1.8% vs 35.6%; p<0.001 and 14.3% vs 29.4%; p = 0.024, respectively). Conversely, the DAVFs located at the tentorium, frontalbasal, foramen magnum and convexity showed a significantly higher risk of intracranial hemorrhage when compared with the lesions located at other regions (33.9% vs 17.8%, p = 0.010; 17.9% vs 6.1%, p = 0.007; 5.4% vs 0.6%, p = 0.043; 16.1% vs 2.2%, p<0.001, respectively). As to the other locations, there was no significant difference between the 2 groups.

DAVFs with arterial feeders from multiple systems showed significantly lower risk of intracranial hemorrhage (67.9% vs 82.8%; p = 0.016). However, no significant higher risk of intracranial hemorrhage was observed in DAVFs with bilateral arterial feeders (41.1% vs 47.8%, p = 0.379). On the other hand, the number of the fistula (single or multiple) or fistula flow velocity (high or low) was not significant correlated with of risk of intracranial hemorrhage in this series (p = 0.162 and 0.167, respectively). As to the variables concerning venous drainage, the DAVFs with superficial cortical venous drainage, deep venous drainage, or occluded venous sinus showed increased risk of intracranial hemorrhage compared with the lesions without them (78.6% vs 40.6%, p<0.001; 32.1% vs 14.4%, p = 0.003; 23.2% vs 11.7%, p = 0.032; respectively). Additionally, DAVFs with higher Borden type also appeared associated with a significantly higher risk of intracranial hemorrhage (Borden type I vs Borden type II vs Borden type III = 80.4% vs 16.1% vs 3.6%, p<0.001).

A multivariate analysis through a logistic regression model was performed considering hemorrhagic occurrence as the dependent variable and the foregoing potential risk factors (p<0.05 in univariate analysis) as covariates. This analysis showed that higher Borden classification (OR 5.880; 95% CI, 3.370–10.257; p<0.001) was the only independent risk factor of intracranial hemorrhage occurrence in our series.

## Discussion

Several previous studies have helped us to identify the risk factors correlated with hemorrhagic occurrence in patients with intracranial DAVF [2, 4, 5, 7–12]. Currently there is only one study investigating both the clinical and angioarchitectural characteristics as the risk factors for hemorrhagic presentation in patients with DAVF [4]. In their study, Singh V et al found that DAVFs with cortical venous drainage, DAVFs in the posterior fossa, male sex, patient age older than 50 years, and focal neurological deficits were independently associated with hemorrhagic presentation in patients with DAVF. In our study, based on a large case series, we analysed the association between the potential clinical and angioarchitectural risk factors and the occurrence of intracranial hemorrhage in patients with DAVF, and found that venous drainage pattern was the only independent risk factor of hemorrhage occurrence.

For demographic factors, previous reports have suggested that sex and age may have a strong relationship with hemorrhage. Singh V et al found that male sex and patient age older than 50 years were independently associated with hemorrhagic presentation in patients with DAVF [4]. However in our study, patient age (including the original age or that was dichotomized by the point of 50 years) was not a potential risk factor for hemorrhagic presentation in univariate analysis. Although male patients showed a higher risk of hemorrhagic presentation in univariate analysis, the multivariate analysis showed sex was not an independent risk factor for hemorrhagic presentation. In our series, 41 patients with cavernous sinus DAVF were female (female vs male = 63.1% vs 36.9%, p<0.001). DAVFs in cavernous sinus are different in several aspects from DAVF involving other locations [14]. Because of the sufficient venous drainage routes of CS, their clinical presentation are known to be benign with a very low risk of intracranial hemorrhage (1/65, 1.5% in our series) [4, 5, 15]. This may be why sex was a risk factor only in univariate analysis. The age effect is more difficult to explain and its correlation with hemorrhagic presentation need to be further studied.

Clinical factors of medical history, including smoking, hypertension, diabetes, alcohol consumption and hyperlipidemia, which are known to affect cerebrovascular diseases of the arterial side, such as intracranial aneurysm, may also be able to affect pathophysiology, structure of vessel wall or hemodynamics of the cerebrovascular diseases on the venous side. Thus, these factors may influence the risk of hemorrhage in cerebrovascular diseases on the venous side. In our study, diabetes mellitus, hyperlipidemia and alcohol consumption are three factors that were firstly investigated as potential risk factors of hemorrhagic occurrence in patients with DAVF. Among them, alcohol consumption was the only risk factor in univariate analysis, but none of these three factors was independent risk factors. Hypertension and smoking are considered as 2 important risk factors for spontaneous intracranial hemorrhage in some cohort studies [16–18]. However, similar to Singh V et al’s study [4], our study shows that both of them were not independent risk factors for hemorrhagic presentation of DAVF, although the prevalence of smoking was significantly higher in the hemorrhagic group.

The number and source of arterial feeders and rate of shunting are some of the theoretical risk factor that may be correlated with hemorrhagic presentation. However, in our study, none of these variables was independently associated with intracranial hemorrhage in our patients. Presence of venous sinus occlusion has been reported by many to be associated with DAVF occurrence [9, 19], also a risk factor for hemorrhagic presentation in patients with DAVF because of the elevated local venous pressure [6, 7, 20]. Although venous sinus occlusion is more common in the hemorrhagic group, it was not independently associated with hemorrhagic presentation in our patients.

In some cohort studies patients with DAVF, those in tentorium, frontalbasal, convexity and foramen magnum location were reported to have a higher risk of intracranial hemorrhage [21–24], as verified in our cohort. In our multivariate analysis, none of them reach statistical significance to independently associate with risk of hemorrhagic presentation. DAVFs in these locations differ from other subtypes because they usually have direct drainage into cortical veins, some with formation of venous ectasia. These characteristics make the lesions prone to intracranial hemorrhage [21–24]. Therefore, lesion location may be only a confounding factor in predicting intracranial hemorrhage and the underlying mechanism may be more associated with the venous drainage pattern of DAVF.

Our study did not identify a new independent risk factor and only confirmed the old finding that cortical venous drainage is independently associated with the risk of hemorrhagic presentation in intracranial DAVFs. Our “negative results” makes this study seems that this study only replicate existing studies and no novel data is presented. However, considering the fact that some clinical and angioarchitectural factors were firstly evaluated as potential risk factor in this study, “negative results” is also a conclusion. We do think that our study, which enrolled 236 cases of DAVFs, is somewhat meaningful in helping the clinicians to understand the hemorrhagic risk factors of DAVF better.

Several limitations of the present report must be noted because of some potential biases. Firstly, our study is a single-center, retrospective study that carries the inherent risk of patient selection bias. This is not a population-based study, but we have selected only patients admitted to our department and performed this study in a retrospective fashion. Thus in fact, we only show risk factors of hemorrhagic presentation in patients with dural DAVFs, but not predictors of it. Moreover, although this is a relatively large case series of patients with DAVFs, with the aim to identify the risk factor of intracranial hemorrhage using logistic regression analysis, the case number in the present study was small. So the results obtained in the present study should be interpreted with caution. To exclude any such limitations in future research, larger prospective studies are required in the future to improve the precision of the present findings and provide more data on this issue for better management of these lesions.

## Conclusions

Venous drainage pattern was the only independent risk factor of intracranial hemorrhage occurrence in our series. The other potential risk factors may be confounding factors in predicting intracranial hemorrhage.

## Supporting Information

S1 File SPSS Statistics Data Document for Statistical Analysis. (SAV)
